# Effects of Aerobic Exercise on Tau and Related Proteins in Rats with the Middle Cerebral Artery Occlusion

**DOI:** 10.3390/ijms21165842

**Published:** 2020-08-14

**Authors:** Sakulrat Mankhong, Sujin Kim, Sohee Moon, Kyoung-Hee Lee, Hyeong-Eun Jeon, Byeong-Hun Hwang, Jong-Won Beak, Kyung-Lim Joa, Ju-Hee Kang

**Affiliations:** 1 Department of Pharmacology, College of Medicine, Inha University, Incheon 22212, Korea; sakulratkulrat@gmail.com (S.M.); sujin2419@hanmail.net (S.K.); moon219@inha.ac.kr (S.M.); 2 Hypoxia-Related Diseases Research Center, College of Medicine, Inha University, Incheon 22212, Korea; 3 Department of Occupational Therapy, Baekseok University, Chungnam 31065, Korea; otprin38@bu.ac.kr; 4 Department of Physical & Rehabilitation Medicine, College of Medicine, Inha University, Incheon 22332, Korea; marine1003@hanmail.net; 5 Industry-Academia Cooperation Group, Baekseok University, Chungnam 31065, Korea; ckgodman@gmail.com (B.-H.H.); marine1003@naver.com (J.-W.B.)

**Keywords:** stroke, acetylated tau, rehabilitation, aerobic exercise, Alzheimer’s disease

## Abstract

Although Alzheimer’s disease (AD)-like pathology is frequently found in patients with post-stroke dementia, little is known about the effects of aerobic exercise on the modifications of tau and related proteins. Therefore, we evaluated the effects of aerobic exercise on the phosphorylation and acetylation of tau and the expressions of tau-related proteins, after middle cerebral artery occlusion (MCAO) stroke. Twenty-four Sprague–Dawley rats with MCAO infarction were used in this study. The rehabilitation group (RG) received treadmill training 40 min/day for 12 weeks, whereas the sedentary group (SG) did not receive any type of training. Functional tests, such as the single pellet reaching task, rotarod, and radial arm maze tests, were performed monthly for 3 months. In ipsilateral cortices in the RG and SG groups, level of Ac-tau was lower in the RG, whereas levels of p-tau_S396_, p-tau_S262_, and p-tau_S202/T205_ were not significantly lower in the RG. Level of phosphorylated glycogen synthase kinase 3-beta Tyr 216 (p-GSK3β_Y216_) was lower in the RG, but levels of p-AMPK and phosphorylated glycogen synthase kinase 3-beta Ser 9 (p-GSK3β_S9_) were not significantly lower. Levels of COX-2 and BDNF were not significantly different between the two groups, while SIRT1 significantly decreased in ipsilateral cortices in RG. In addition, aerobic training also improved motor, balance, and memory functions. Rehabilitation with aerobic exercise inhibited tau modification, especially tau acetylation, following infarction in the rat MCAO model, which was accompanied with the improvement of motor and cognitive functions.

## 1. Introduction

Pathologic accumulation of tau is a characteristic feature of a group of neurodegenerative disorders classified as tauopathy, of which Alzheimer’s disease (AD) is the most common [1]. Several studies have investigated the posttranslational modifications of tau toxicity, including phosphorylation, ubiquitination, methylation, and acetylation [2,3,4]. Tau acetylation has been known to trigger tau aggregation, implicating its effects in the pathogenic cascade leading to tau pathology [5]. Tau pathology was observed in neurons and glia, which might be associated with cerebrovascular damage after ischemic stroke and may play a critical element in post-stroke dementia [6].

Our previous study on a photochemically induced thrombosis (PIT) infarction model showed that tau phosphorylation and acetylation were increased after stroke. Furthermore, the expression levels of tau-related proteins such as tau kinases, α-synuclein (α-syn), and proteins involved in neuroinflammation were differentially regulated after stroke. α-syn mediates ischemic brain damage [7] and is implicated in neurodegenerative changes in neuronal and glial cells [8,9].

In addition, task-specific training (TST) was found to reduce the phosphorylation and acetylation of tau and expressions of tau-related proteins after stroke [10]. Majority of ischemic human strokes result from an occlusion of a major cerebral artery by thrombosis or embolism [11]. In human stroke, reperfusion by thrombolytic drugs produce ischemic penumbra, which is potentially a salvageable area where neural plasticity takes place with rehabilitation [12]. While PIT infarction makes cell apoptosis and clear infarction margins with no penumbra, the reperfusion process followed by occlusion with MCAO could make a penumbra area, mimicking the human ischemic stroke pathophysiology more [13]; a recent study reported that exercise improved the expression of neuroplasticity proteins including tau and brain-derived neurotrophic factor (BDNF) in a MCAO model of rats [14].

Aerobic exercise has shown to inhibit amyloid beta protein and hyperphosphorylated tau protein accumulations in the AD mouse model [15,16]. In a recent randomized controlled study, aerobic exercise in early AD was found to be associated with benefits in functional ability [17]. Cardiorespiratory fitness, which was obtained by aerobic exercise, was associated with improved memory performance and reduced hippocampal atrophy [17]. Although AD-like disease is frequently found in patients with post-stroke dementia [18], little is known about the effects of aerobic exercise on the modifications of tau and related proteins. Therefore, this study aimed to evaluate the effect of aerobic exercise on the phosphorylation and acetylation of tau and the expressions of tau-related proteins after MCAO stroke.

## 2. Result

### 2.1. Comparing the Levels of Tau Phosphorylation, Acetylation, and Tau-Related Proteins between Ipsilateral and Contralateral Cortices of SG Groups after Three-Month Aerobic Exercise

**Figure 1:**
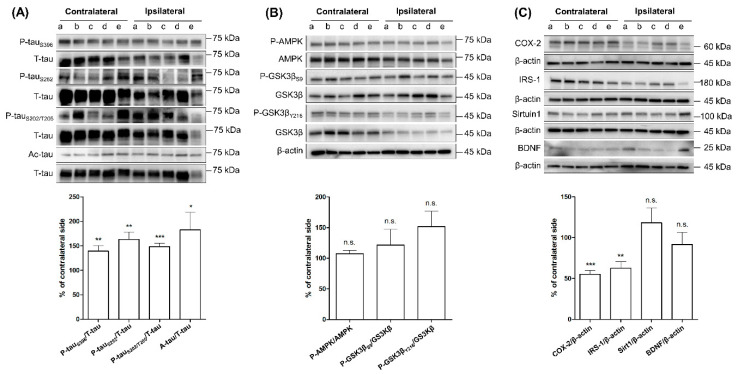
Phosphorylation and acetylation of tau and related proteins in ipsilateral and contralateral cortices in SG at three months after MCAO. Comparison of the levels of protein in ipsilateral cortex with matched contralateral cortex (indicated by the same alphabet) (*n* = 9 per group). (**A**) Phosphorylated tau and acetylated tau. (**B**) p-AMPK, p-GSK3βS9, and p-GSK3βY216. (**C**) COX-2, IRS-1, SIRT1, and BDNF. * *p* < 0.05, ** *p* < 0.01, and *** *p* < 0.001 vs. paired contralateral region by one-sample *t*-test. Vertical bars in graphs indicate standard deviations. T-tau—total tau; p-AMPK—Phosphorylated 5′-AMP—activated protein kinase; p-GSK3β—phosphorylated glycogen synthase kinase-3 beta; COX-2—Cyclooxygenase 2; IRS-1—Insulin receptor substrate 1; SIRT1—Sirtuin 1; BDNF—Brain-derived neurotrophic factor.

**Figure 2:**
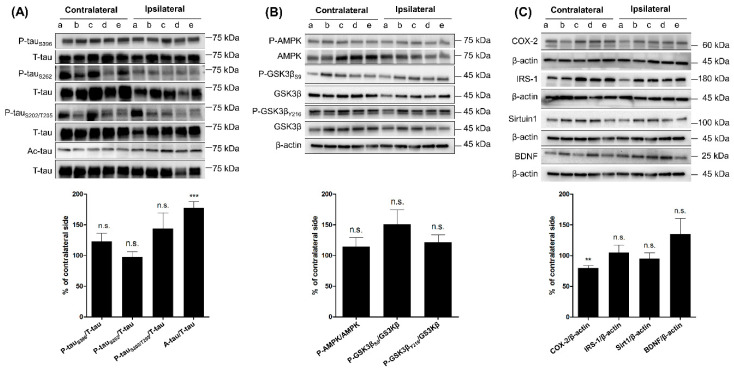
Effect of three-month aerobic exercise on the phosphorylation and acetylation of tau and related proteins in ipsilateral and contralateral cortices in RG. Comparison of the levels of protein in ipsilateral cortex with matched contralateral cortex (indicated by the same alphabet) (*n* = 9 per group). (**A**) Phosphorylated tau and acetylated tau. (**B**) p-AMPK, p-GSK3βS9, and p-GSK3βY216. (**C**) COX-2, IRS-1, SIRT1, and BDNF. * *p* < 0.05, ** *p* < 0.01, and *** *p* < 0.001 vs. paired contralateral region by one-sample t-test. Vertical bars in graphs indicate standard deviations. T-tau—total tau; p-AMPK—Phosphorylated 5′-AMP-activated protein kinase; p-GSK3β—phosphorylated glycogen synthase kinase-3 beta; COX-2—Cyclooxygenase 2; IRS-1—Insulin receptor substrate 1; SIRT1—Sirtuin 1; BDNF—Brain-derived neurotrophic factor.

**Figure 3:**
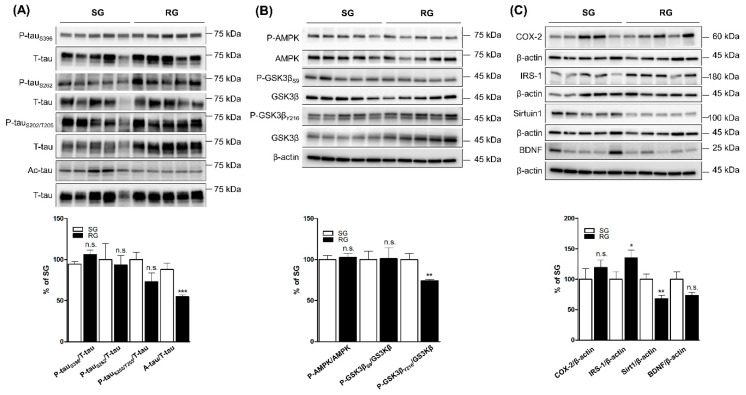
Effect of three-month aerobic exercise on the phosphorylation and acetylation of tau and related proteins. Comparison of levels of proteins in ipsilateral cortices in the RG (black bar) and SG (white bar) (*n* = 9 per group). (**A**) Phosphorylated tau and acetylated tau. (**B**) p-AMPK, p-GSK3β_S9_, and p-GSK3β_Y216_. (**C**) COX-2, IRS-1, SIRT1, and BDNF. * *p* < 0.05, ** *p* < 0.01, and *** *p* < 0.01 vs. SC group by Mann–Whitney U test. Vertical bars in graphs indicate standard deviations. RG—Rehabilitation group; SG—Sedentary control group; T-tau—total tau; p-AMPK—Phosphorylated 5′-AMP-activated protein kinase; p-GSK3β—phosphorylated glycogen synthase kinase-3 beta; COX-2—Cyclooxygenase 2; IRS-1—Insulin receptor substrate 1; SIRT1—Sirtuin 1; BDNF—Brain-derived neurotrophic factor.

We compared the expression levels of protein in two ways. First, we compared the levels between contralateral and ipsilateral cortices in SG and RG (Figure 1 and Figure 2) and compared the levels in ipsilateral cortices of SG with those of RG (Figure 3). The expression levels of p-tau_S396_, p-tau_S262_, p-tau_S202/T205_, and Ac-tau in ipsilateral cortex (IC) significantly increased compared to the contralateral cortex (CC) (Figure 1A). Levels of p-AMPK, p-GSK3β_S9_, and p-GSK3β_Y216_ were not significantly different between IC and CC (Figure 1B). Levels of COX-2 significantly decreased in IC, while levels of SIRT1 and BDNF were not different between IC and CC (Figure 1C).

### 2.2. Comparing the Levels of Tau Phosphorylation, Acetylation, and Tau-Related Proteins between Ipsilateral and Contralateral Cortices of RG Groups after Three-Month Aerobic Exercise

The expression levels of p-tau_S396_, p-tau_S262_, and p-tau_S202/T205_ in IC were not significantly different compared to CC (Figure 2A); however, the level of Ac-tau significantly increased in IC (Figure 2A). Levels of p-AMPK, p-GSK3β_S9_, and p-GSK3β_Y216_ were not significantly different between IC and CC (Figure 2B). Levels of SIRT1 and BDNF were not significantly different between IC and CC, while level of COX-2 significantly decreased in IC (Figure 2C).

### 2.3. Comparing the Levels of Tau Phosphorylation, Acetylation and Tau-Related Proteins in Ipsilateral Cortices of RG with SG Groups after Three-Month Aerobic Exercise

In ipsilateral cortices in the RG and SG groups, level of Ac-tau was lower in the RG, whereas levels of p-tau_S396_, p-tau_S262_, and p-tau_S202/T205_ were not significantly lower in the RG (Figure 3A). Level of phosphorylated glycogen synthase kinase 3-beta Tyr 216 (p-GSK3β_Y216_) was lower in the RG, but levels of p-AMPK and phosphorylated glycogen synthase kinase 3-beta Ser 9 (p-GSK3β_S9_) were not significantly lower (Figure 3B). Levels of COX-2 and BDNF were not significantly different between the two groups; however, SIRT1 significantly decreased in the ipsilateral cortices in RG. Meanwhile, IRS-1 significantly increased in the ipsilateral cortices in RG (Figure 3C).

### 2.4. Improvements in Motor and Balance Functions Caused by Aerobic Training (12 Rats per Group)

**Figure 4:**
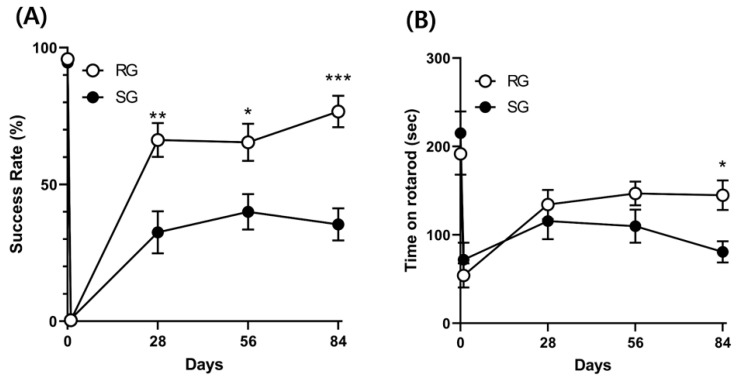
Recovery of fine motor and balance function during the post-ischemic period. (**A**) Improvements in fine motor function as determined by success rates (%) of results of the single pellet reaching (SPR) test (*n* = 12 per group; F = 7.875; *p* = 0.001). (**B**) Improvements in balance function as determined by the rotarod test (F = 2.701; *p* = 0.03). Vertical bars indicate standard errors. * *p* < 0.05, ** *p* < 0.01, and *** *p* < 0.01 vs. SG group by Mann–Whitney U test.

Both single pellet reaching (SPR) success rates and rotarod test results improved over time in the RG and SG groups. However, repeated ANOVA showed that aerobic training significantly improved SPR success rates and rotarod test results over time in comparison with the control group (F = 7.875; *p* = 0.001 and F = 2.701; *p* = 0.03, respectively) (Figure 4).

### 2.5. Improvements in Memory Functions Caused by Aerobic Training (12 Rats per Group)

**Figure 5:**
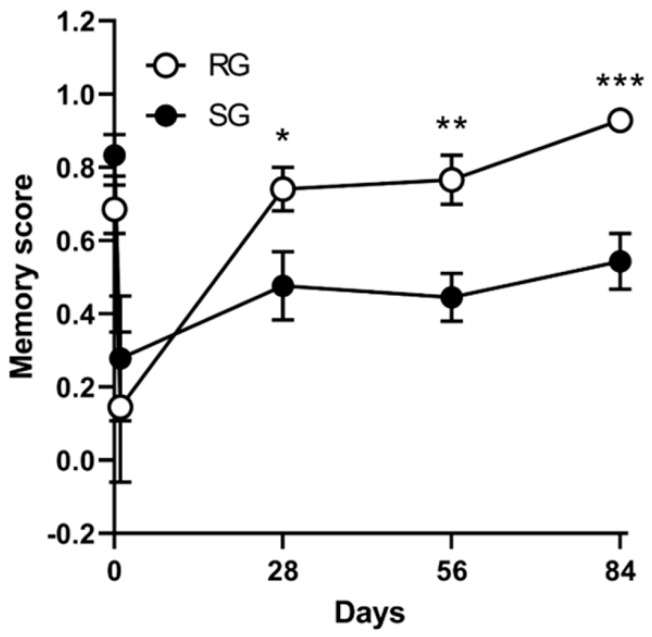
Improvements in memory function as determined using the radial arm maze test memory scores (*n* = 12 per group; F = 3.365; *p* = 0.04). Vertical bars indicate standard errors. * *p* < 0.05, ** *p* < 0.01, and *** *p* < 0.01 vs. SG group by Mann–Whitney U test.

Radial maze results improved over time in both RG and SG groups. However, repeated ANOVA showed aerobic training significantly improved memory scores over time in comparison with the control group (F = 3.365; *p* = 0.04) (Figure 5).

## 3. Discussion

Given that ischemic brain stroke induces tau hyperphosphorylation due to the activation of a variety of tau kinases [19], inhibition of tau pathology and subsequent neurotoxicity may be a target for mitigating the course of post-stroke dementia. The effect of aerobic exercise on tau proteins has been evaluated with transgenic mice expressing human pathogenic tau gene or presenilin 2 genes [20,21,22]. However, the effects of aerobic exercise on the expression of tau protein and tau-related proteins have not been intensively studied in vivo after ischemia-reperfusion brain stroke. Our previous study found that four weeks of TST greatly improved fine motor function and suppressed p-tau expression probably through the p-AMPK and Akt-mTORC1-p70S6K pathway after PIT stroke [10].

In this study, we changed the mode of exercise and model of brain stroke from TST to aerobic training and PIT to MCAO model, respectively, to evaluate the difference. Furthermore, for evaluating the long-term effects of exercise, we expanded the period of exercise from 4 weeks to 12 weeks. We found that 12-week aerobic training significantly reduced Ac-tau in the IC of RG, as compared to the level of SG (Figure 3). However, the levels of phosphorylated tau (p-tau_S396_, p-tau_S262_, and p-tau_S202/T205_) were not significantly different between RG and SG after aerobic training. The tau hyperphosphorylation is controlled by many tau kinases with different specific phosphorylation sites [23]. After aerobic exercise, the levels of p-AMPK and p-GSK3β_S9_ in the IC of RG were not significantly different from that of those in SG. AMPK is known to phosphorylate tau at Ser^396^ epitopes [24]. Our results showed that p-tau_S396_ and associated tau kinase, p-AMPK, were not controlled by aerobic training after MCAO stroke. Although the level of p-GSK3β_S9_, which is known to have inhibitory effect on tau phosphorylation, was not significantly different and the level of GSK3β_Y216_ (phosphorylation with increased activity of GSK3β) significantly decreased between the two groups after aerobic training. GSK3β_Y216_ is another tau kinase with respect to the generation of pathological phosphoepitope (e.g., Ser396) [25]. Since p-tau_S396_ did not significantly change with 12 weeks of aerobic training, other pathologic phosphoepitopes induced by GSK3β_Y216_ could be evaluated with further study. Another possibility of the mismatch between the levels of phosphorylated tau and p-GSK3βY216 could be the long-term effects of aerobic training on brain metabolism, since GSK3β plays a crucial role in metabolism [26]. In fact, the level of IRS-1 in the IC of RG was significantly higher than that of SG, indicating that a 12-week aerobic training may regulate brain metabolism as well as tau protein modification.

Recent studies on spontaneous old rat models and transgenic mice models of AD showed a protective effect of aerobic exercise on tau phosphorylation and related tau kinases [15,27,28]. Although previous investigations with rat stroke models showed increased level of hyperphosphorylation of tau [29,30,31,32], there have been only a few studies evaluating the effect of aerobic training on tau phosphorylation and related proteins after stroke so far. One study of old P301 transgenic mice showed that 12 weeks of treadmill training had a beneficial effect on tauopathy [15]. In that study, p-tau reduction was more prominent in spinal cord versus brain. Further, in the brain, the p-tau was more prominent in the hippocampus rather than the cortex [15]. The most recent study demonstrated that mild and intense exercise training for up to 28 days reduced infarct volume and improved brain function in the rat MCAO model [14]. Interestingly, the levels of tau protein and brain-derived neurotropic factor (BDNF) were higher in the mild aerobic exercise group than the intense training group, which suggests the variability in the effects of exercise on tau-related pathology according to the intensity, type, and duration of exercise [14].

In our study, there was no difference in p-tau between RG and SG. Further study with a larger study group and region-specific tau investigation, such as hippocampus and spinal cord, is warranted.

In another pathologic feature of tau pathogenesis, Ac-tau was significantly lower in the IC of RG compared to that of SG in this study (Figure 3). Acetylation of tau promotes tau hyperphosphorylation, tau aggregation, and tangle formation [2,3,5]. Although Ac-tau has been focused on as a disease-modifying target for drug discovery in tauopathies [3], there have been no studies evaluating the effect of aerobic training on Ac-tau in stroke and AD model. To our knowledge, this is the first study showing the protective role of aerobic training in tau-related neurodegeneration after stroke. It is only an aerobic training-mediated effect because Ac-tau did not significantly decrease after TST in our previous study [10]. SIRT1 has been found to reduce Ac-tau [33]. However, the inhibitory effect of aerobic training on Ac-tau might be independent of SIRT1-mediated mechanism, because the level of SIRT1 in the IC of RG was significantly lower than that of SG. The inhibitory effect of aerobic training on Ac-tau might be mediated by other unknown acetylation–deacetylation mechanisms. When we compared IC to CC, increased levels of Ac-tau were observed in IC in both RG and SG (Figure 1 and Figure 2). These findings suggest that the tau pathology through acetylation and subsequent neurotoxic effect may be sustainable even after three months of aerobic training. Hence, compared to tau phosphorylation, the longer effect of Ac-tau by ischemia-reperfusion brain stroke should be focused for therapeutic target after stroke.

In this study, the level of COX-2 was not different between the two groups. Regular aerobic exercise has been known to reduce peripheral inflammation and COX-2 levels [34]; however, the effects of aerobic exercise on COX-2 expression in the brain have not been well investigated. A recent study reported that brain COX-2 levels increased after endurance training, and its expression positively correlated with memory functions [34,35]. In another study, treadmill training was found to have time-dependent up and down regulatory effects on the COX-2 pathway [36]. Further studies with time-dependent COX-2 level after stroke and its relationship with tauopathy or neuroinflammation are warranted.

In this study, both fine motor function by the single pellet reaching task and balance control by the rotarod test were improved by aerobic exercise, which was more prominent than natural recovery in SG. Moreover, memory score by the radial maze test was improved more rapidly by aerobic exercise. The radial arm maze test measures spatial working memory. Working memory deficits are a recognized feature of AD [37]. They are commonly attributed to executive impairment and assumed to be related to frontal lobe dysfunction. Since balance and fine motor functions require executive functioning, working memory is closely related to balance and fine motor control [38,39]. Our study presented the beneficial effects of long-term aerobic exercise on both spatial working memory as well as balance and fine motor functions, which is consistent with our previous study on a focal ischemia PIT model [10]. The functional improvement by aerobic training in the stroke model could not be fully explained by changes of tau-related proteins in this study. Tau-related pathogenesis is well-known neurodegenerative changes in various diseases, including stroke [1,6]; however, molecular mechanisms underlying the chronological improvement of motor and memory functions by aerobic training should be further elucidated.

There are several limitations in this study. First, we evaluated only some p-tau and related proteins in this study; more types of p-tau and related proteins need to be evaluated in future studies. Second, multiple cognitive domains were not evaluated, except working memory. Therefore, further studies using multiple cognitive behavioral tests should be conducted. Third, since the number of rats in each group was relatively small, inter- and intra-group variations limited our abilities to interpret the results. Finally, cause–effect relationships between tau and other AD-related proteins were not evaluated. For this, transgenic mice presenting overexpression of AD-related pathogenic proteins (e.g., 5XFAD mice) can be a good model for evaluating the effects of different types of rehabilitation on post-stroke neurodegeneration and cognitive dysfunction.

In conclusion, rehabilitation with aerobic exercise inhibited tau modification, especially tau acetylation, following infarction in the rat MCAO model, which was accompanied with the improvement of motor and cognitive functions.

## 4. Materials and Methods

### 4.1. Experimental Animals

Male Sprague–Dawley rats (*n* = 24), 8 weeks old and weighing 250–300 g were obtained from Orientbio Inc. (Seungnam, Korea). The rats were housed 2 per cage with free access to food and water in a temperature (22 ± 2 °C) and humidity-controlled (45–55%) environment by 12:12-h light/dark cycle (with lights on at 8:00 a.m.). All experiments were performed in accordance with the Institutional Animal Care and Use Committee of Inha University (number: INHA 20160905-436). Twenty-four rats underwent MCAO ischemic surgery and were randomly allocated to a rehabilitation group (RG, *n* = 12) or a sedentary control group (SG, *n* = 12) for 12 weeks. Behavioral tests were performed monthly for 3 months. Twelve weeks after stroke, these 24 rats were sacrificed. The expressions of phosphorylated and acetylated tau and of tau-related proteins in ischemic cortices were then evaluated. The infarction areas of both groups were compared with random samples (*n* = 3 for each group). The infarction area did not differ, and no infarct volume difference was observed between the two groups.

### 4.2. Induction of MCAO Infarction

The rats were anesthetized with isoflurane (4% for surgical induction, 1.75% for maintenance) in NO_2_: O_2_ (70%:30%) and subjected to MCAO operation [40]. Body temperature was monitored continuously with a rectal probe and maintained at 37.5 °C ± 0.5 °C using a heating pad. The opposite side of the dominant hand middle cerebral artery (MCA) was established by proximal occlusion of the MCA using a 4–0 silicone-coated nylon monofilament (5 mm length of the coated end with a diameter of 0.35 mm). After a 90-min occlusion, reperfusion was accomplished by careful withdrawal of the monofilament and the rats were returned to their cages [40]. Neurological deficits examination was conducted at 3 h and 1 day after MCAO by an investigator who was blind to the experiment design according to the five-point scale described previously by Longa et al. [41] as follows: 0 indicated no neurological deficit; 1—mild focal neurological deficit (contralateral forelimb flexion upon lifting the animal tail); 2—moderate focal neurological deficit (circling to the contralateral side when crawling forward); 3—severe focal deficit (falling into the contralateral side when crawling forward); 4—no spontaneous crawling with a depressed level of consciousness or death. Only rats with neurological scores of 1 to 3 were considered successful models and used in the current study [41].

### 4.3. Western Blot

Isolated cerebral cortices were homogenized on ice in radioimmunoprecipitation assay buffer (50 mM Tris-HCl, pH 7.5, 1% Triton X-100, 150 mM NaCl, 1% sodium deoxycholate, 2 mM EDTA, and 0.1% SDS) containing proteases inhibitors and a phosphatase inhibitor cocktail [42]. Total protein extracts (15 μg) were separated by 4–20% SDS-PAGE and subsequently, transferred to PVDF membranes (Millipore, USA). Membranes were incubated in blocking buffer containing 5% non-fat dried milk or 3–5% bovine serum albumin at room temperature for 1 h and then, probed with primary antibodies (data show in Table 1) for 2 or 16 h at RT or 4 °C, respectively. Specific protein signals were visualized using an enhanced chemiluminescent substrate (Thermo Scientific, Rockford, IL, USA). For quantitative analysis, band signal intensities were analyzed by densitometry using image analysis software Quantity One^®^ 4.6 (Bio-Rad, Laboratories, Hercules, CA, USA). Levels of Sirtuin-1 (SIRT1), Brain derived neurotrophic factor (BDNF), and COX-2 were determined using β-actin as a loading control. Ratios of phosphorylated or acetylated protein signals versus total protein signals were calculated for phosphorylated proteins: phosphorylated 5′-AMP-activated protein kinase (p-AMPK), phosphorylated tau, phosphorylated glycogen synthase kinase 3 (p-GSK3), and acetylated tau (Ac-tau).

### 4.4. The Single Pellet Reaching (SPR) Test

The apparatus used was made of clear Plexiglas (40 × 45 × 13.1 cm) with a 1 cm wide slit in the middle of the front wall. The food shelf was attached behind this slit [43]. Rats were evaluated for paw dominance and were administered 20 pellets per day for 3 weeks before surgery. Dominant limbs were determined when a rat grasped a sucrose pellet (Bio-Serve, Frenchtown, NJ, USA) at least 16 times during 20 attempts with one forelimb. After we performed MCAO opposite to dominant paw side, the SPR test was performed 1 day after stroke and then, monthly for 3 months. During the SPR test, a pellet was placed opposite to the preferred paw to prevent the use of the non-dominant paw. Successful reach was defined as an animal grasping a food pellet, bringing it into its cage, and consuming it without dropping the pellet. Success rate was defined as the percentage of successful reaches, as defined by the following formula [44]:

$$Success\;rate\;\left(\%\right)=\frac{Number\;of\;successful\;reached\;\times 100}{20}$$

### 4.5. Rotarod Test

Balance skills, motor performance, and muscle strength were evaluated on an accelerating rotating rod (JD-A-09, JEUNG-DO B&P, Seoul, Korea). All rats were trained over 3 days to stay on the rotarod before surgery, and times spent on the revolving rod before surgery were used as baseline values. During the training period and after surgery, rotarod speed was increased from 10 to 60 revolutions per minute (rpm). The cut-off time was set at 300 s. Each animal underwent three trials per day with 30 min inter-trial intervals. Time to maintain balance on the revolving rod was measured in seconds [45]. Rotarod testing was performed 1 day after surgery and then, monthly for 3 months.

### 4.6. The Radial Maze Test

We used an 8-arm radial maze to assess spatial working memory. The apparatus was made of plastic and had an octagonal central platform (34 cm diameter) and eight equally spaced radial arms (87 cm long, 10 cm wide). Each arm contained a food cup (5 mm deep and 3 cm in diameter). Cups were glued to the surfaces of the arms ~1 cm from arm ends. Entrance to the arms could be blocked by guillotine doors 24.5 cm high and 11 cm wide. The entire apparatus was surrounded by a 24.5 cm high transparent wall and elevated 43 cm above floor level. Furthermore, the apparatus was surrounded by various extra-maze cues, including a screen, a brightly colored poster, and a clock [46]. This test involved a training and a test phase. Before the training phase, four arms were randomly chosen and blocked, but no more than two adjacent arms were blocked in any trial. The unblocked arms were baited with grain reward pellets. During the training phase, rats were allowed 5 min to enter arms and retrieve grain pellet rewards from baited arms. After a 5 min inter-trial interval, the test phase was initiated. During this phase, all eight arms were opened and previously blocked arms were baited with grain pellets. A rat was placed inside the maze and the number of arm entries was recorded [46]. Before surgery, rats were habituated to the maze for 3 days. After habituation, all 24 rats were tested just before surgery (baseline). After surgery, testing was performed 1 day after infarction and then, monthly for 3 months. The rats acquired information on maze arms during the training phase, retained this information during the intervening period, and recalled it during the test phase [46]. Numbers and durations of arm entries were recorded by computerized monitoring. An entry to any arm that did not contain a food pellet was regarded as an error. The number of errors made during the training and test phases (training- and test-phase errors, respectively) were recorded. Memory scores were calculated as follows [47]:

$$Memory\;score=\frac{\left(correct\;arm\;entry\right)-\left(incorrect\;arm\;entry\right)}{\left(correct\;arm\;entry\right)+\left(incorrect\;arm\;entry\right)}$$

### 4.7. Treadmill Aerobic Exercise Training

A motor-driven treadmill (JD-A-07-RA5, JEUNG-DO B&P, Seoul, Korea) was used for aerobic exercise training. Before the MCAO infarction surgery, all rats were adapted to running on the treadmill for 10 min/day over a 3-day acclimatization period. Three days after surgery, rats in the RG were trained to run on the treadmill at 20 m/min for 30 min/day, 5 days/week for 12 weeks at an inclination of 0^0^ [48]. Correspondingly, rats from the control group were left on the treadmill without running for the same time.

### 4.8. Statistical Analysis

Relative expression levels of proteins in ipsilateral versus contralateral sides were compared using the one-sample t-test. For unpaired samples, the nonparametric Mann–Whitney U test was used to compare the two study groups. Western blot data were analyzed using GraphPad Prism version 6.0 (GraphPad Software Inc., San Diego, CA, USA). Single pellet reaching task, rotarod test, and radial maze results were analyzed using repeated measures ANOVA and the Mann–Whitney U test in SPSS ver. 19.0 (SPSS Inc., Chicago, IL, USA). Results were presented as means ± standard errors or standard deviations, and statistical significance was accepted for *p* values < 0.05.

## Figures and Tables

**Table 1:** List of antibodies used in present study.

Antibodies	Dilution	Manufacturers	Source
Phospho-tau (Ser396)	1:1000	Santa Cruz (sc-12414)	Rabbit
Phospho-tau (Ser262)	1:500	Invitrogen (44-750G)	Rabbit
Phospho-tau (Ser202, Thr205)	1:1000	Thermo Scientific (MN1020)	Mouse
Ac-tau	1:500	Anaspec (As56077)	Rabbit
T-tau (tau-5)	1:1000	Thermo Scientific *AHB0042)	Mouse
Phospho-AMPKα (Thr172)	1:1000	Cell signaling (2535)	Rabbit
AMPKα	1:1000	Cell signaling (2532)	Rabbit
Phospho-GSK3βS9	1:1000	Cell signaling (9336)	Rabbit
Phospho-GSK3βY216	1:1000	Santa Cruz (sc-135653)	Rabbit
GSK3β	1:1000	Cell signaling (9315)	Rabbit
COX-2	1:1000	Abcam (Ab15191)	Rabbit
IRS-1	1:1000	Cell signaling (2382)	Rabbit
Sirtuin1	1:1000	Cell signaling (9475)	Rabbit
BDNF	1:1000	Santa Cruz (sc-65514)	Mouse
β-actin	1:1000	Pro-sci (3779)	Rabbit
